
JOURNAL INFORMATION
==============================
NLM Title Abbreviation: Sci Pharm
Journal ID: Sci Pharm
NLM Journal ID: 0026251
Journal ID: Scientia Pharmaceutica

Scientia Pharmaceutica

ISSN: 0036-8709
EISSN: 2218-0532
Publisher: Österreichische Apotheker-Verlagsgesellschaft, m. b. H.

ARTICLE INFORMATION
==============================
PMCID: PMC3002817
Article ID: scipharm.2010.78.555
Article version: 1
Subjects: Abstracts of the 8th Central European Symposium on Pharmaceutical Technology (CESPT), Satellite Symposium: 4th International Graz Congress on Pharmaceutical Engineering, September 16th-18th, Graz, Austria

Oral Presentations (LDD, LPES, LNM, LPAT, LNT, LPPT, LPM)

Electronic publication date: 2010 Sep 30
Print publication date: 2010 Jul-Sep
Volume: 78
Issue: 3
First page: 555
Last page: 589
Copyright: © author(s); licensee Österreichische Apotheker-Verlagsgesellschaft m. b. H., Vienna, Austria.
Copyright year: 2010
License: This is an Open Access article distributed under the terms of the Creative Commons Attribution License (http://creativecommons.org/licenses/by/3.0/), which permits unrestricted use, distribution, and reproduction in any medium, provided the original work is properly cited.
License URL: https://creativecommons.org/licenses/by/3.0/

==============================
JOURNAL INFORMATION
==============================
NLM Title Abbreviation: Sci Pharm
Journal ID: Sci Pharm
NLM Journal ID: 0026251
Journal ID: Scientia Pharmaceutica

Scientia Pharmaceutica

ISSN: 0036-8709
EISSN: 2218-0532
Publisher: Österreichische Apotheker-Verlagsgesellschaft, m. b. H.

ARTICLE INFORMATION
==============================
DOI: 10.3797/scipharm.cespt.8.LDD01
Article ID: scipharm.2010.78.555a
Subjects: Conference abstract LDD01

Development of Probiotic Formulations Containing Shellac

Stummer S.
Salar-Behzadi S.
Viernstein H.
Department of Pharmaceutical Technology and Biopharmaceutics, University of Vienna, Althanstraße 14, 1090 Vienna, Austria
E-mails: stefanie.stummer@univie.ac.at (S. Stummer), sharareh.salar-behzadi@univie.ac.at (S. Salar-Behzadi)
Electronic publication date: 2010 Sep 30
Print publication date: 2010 Jul-Sep
Volume: 78
Issue: 3
First page: 555
Last page: 555
Copyright: © author(s); licensee Österreichische Apotheker-Verlagsgesellschaft m. b. H., Vienna, Austria.
Copyright year: 2010
License: This is an Open Access article distributed under the terms of the Creative Commons Attribution License (http://creativecommons.org/licenses/by/3.0/), which permits unrestricted use, distribution, and reproduction in any medium, provided the original work is properly cited.

JOURNAL INFORMATION
==============================
NLM Title Abbreviation: Sci Pharm
Journal ID: Sci Pharm
NLM Journal ID: 0026251
Journal ID: Scientia Pharmaceutica

Scientia Pharmaceutica

ISSN: 0036-8709
EISSN: 2218-0532
Publisher: Österreichische Apotheker-Verlagsgesellschaft, m. b. H.

ARTICLE INFORMATION
==============================
DOI: 10.3797/scipharm.cespt.8.LDD02
Article ID: scipharm.2010.78.556
Subjects: Conference abstract LDD02

Non-Invasive Oligonucleotide Delivery System Based on a Thiolated Polymer: Development and In Vitro Evaluation

Martien R. 1 2
Hoyer H. 2
Perera G. 2
Bernkop-Schnürch A. 2
1 Department of Pharmaceutics, Faculty of Pharmacy, Gadjah Mada University, Sekip Utara, 55281 Yogyakarta, Indonesia
2 Department of Pharmaceutical Technology, Institute of Pharmacy, Leopold-Franzens-University of Innsbruck, Innrain 52, 6020 Innsbruck, Austria
E-mail: RonnyMartien@ugm.ac.id (R. Martien)
Electronic publication date: 2010 Sep 30
Print publication date: 2010 Jul-Sep
Volume: 78
Issue: 3
First page: 556
Last page: 556
Copyright: © author(s); licensee Österreichische Apotheker-Verlagsgesellschaft m. b. H., Vienna, Austria.
Copyright year: 2010
License: This is an Open Access article distributed under the terms of the Creative Commons Attribution License (http://creativecommons.org/licenses/by/3.0/), which permits unrestricted use, distribution, and reproduction in any medium, provided the original work is properly cited.

JOURNAL INFORMATION
==============================
NLM Title Abbreviation: Sci Pharm
Journal ID: Sci Pharm
NLM Journal ID: 0026251
Journal ID: Scientia Pharmaceutica

Scientia Pharmaceutica

ISSN: 0036-8709
EISSN: 2218-0532
Publisher: Österreichische Apotheker-Verlagsgesellschaft, m. b. H.

ARTICLE INFORMATION
==============================
DOI: 10.3797/scipharm.cespt.8.LDD03
Article ID: scipharm.2010.78.557
Subjects: Conference abstract LDD03

Microcarriers for Controlled Local Delivery of Mupirocin: Preparation and Characterisation

Dürrigl M. 1
Hafner A. 2
Filipović-Grčić J. 2
1 PLIVA Croatia Ltd., Generics Research and Development, Zagreb, Croatia
2 Dept. of Pharmaceutics, Faculty of Pharmacy and Biochemistry, University of Zagreb, Zagreb, Croatia
E-mails: marjana.durrigl@pliva.com (M. Dürrigl), ahafner@pharma.hr (A. Hafner), jfilipov@pharma.hr (J. Filipović-Grčić)
Electronic publication date: 2010 Sep 30
Print publication date: 2010 Jul-Sep
Volume: 78
Issue: 3
First page: 557
Last page: 557
Copyright: © author(s); licensee Österreichische Apotheker-Verlagsgesellschaft m. b. H., Vienna, Austria.
Copyright year: 2010
License: This is an Open Access article distributed under the terms of the Creative Commons Attribution License (http://creativecommons.org/licenses/by/3.0/), which permits unrestricted use, distribution, and reproduction in any medium, provided the original work is properly cited.

JOURNAL INFORMATION
==============================
NLM Title Abbreviation: Sci Pharm
Journal ID: Sci Pharm
NLM Journal ID: 0026251
Journal ID: Scientia Pharmaceutica

Scientia Pharmaceutica

ISSN: 0036-8709
EISSN: 2218-0532
Publisher: Österreichische Apotheker-Verlagsgesellschaft, m. b. H.

ARTICLE INFORMATION
==============================
DOI: 10.3797/scipharm.cespt.8.LDD04
Article ID: scipharm.2010.78.558
Subjects: Conference abstract LDD04

New Hierarchically Organized Systems for Delivery

Glatter O.
Kulkarni C.
Chemelli A.
Department of Chemistry, Karl-Franzens University, Graz, Austria
E-mail: otto.glatter@uni-graz.at (O. Glatter)
Electronic publication date: 2010 Sep 30
Print publication date: 2010 Jul-Sep
Volume: 78
Issue: 3
First page: 558
Last page: 558
Copyright: © author(s); licensee Österreichische Apotheker-Verlagsgesellschaft m. b. H., Vienna, Austria.
Copyright year: 2010
License: This is an Open Access article distributed under the terms of the Creative Commons Attribution License (http://creativecommons.org/licenses/by/3.0/), which permits unrestricted use, distribution, and reproduction in any medium, provided the original work is properly cited.

JOURNAL INFORMATION
==============================
NLM Title Abbreviation: Sci Pharm
Journal ID: Sci Pharm
NLM Journal ID: 0026251
Journal ID: Scientia Pharmaceutica

Scientia Pharmaceutica

ISSN: 0036-8709
EISSN: 2218-0532
Publisher: Österreichische Apotheker-Verlagsgesellschaft, m. b. H.

ARTICLE INFORMATION
==============================
DOI: 10.3797/scipharm.cespt.8.LDD05
Article ID: scipharm.2010.78.559
Subjects: Conference abstract LDD05

Targeted PLGA-Microparticles as a Novel Concept for Treatment of Lactose Intolerance

Wang X. #
Ratzinger G. #
Wirth M.
Gabor F.
Department of Pharmaceutical Technology and Biopharmaceutics, Faculty of Life Sciences, University of Vienna, Vienna, Austria
# These authors equally contributed to this work.

E-mails: xueyan.wang@univie.ac.at (X. Wang), franz.gabor@univie.ac.at (F. Gabor)
Electronic publication date: 2010 Sep 30
Print publication date: 2010 Jul-Sep
Volume: 78
Issue: 3
First page: 559
Last page: 559
Copyright: © author(s); licensee Österreichische Apotheker-Verlagsgesellschaft m. b. H., Vienna, Austria.
Copyright year: 2010
License: This is an Open Access article distributed under the terms of the Creative Commons Attribution License (http://creativecommons.org/licenses/by/3.0/), which permits unrestricted use, distribution, and reproduction in any medium, provided the original work is properly cited.

JOURNAL INFORMATION
==============================
NLM Title Abbreviation: Sci Pharm
Journal ID: Sci Pharm
NLM Journal ID: 0026251
Journal ID: Scientia Pharmaceutica

Scientia Pharmaceutica

ISSN: 0036-8709
EISSN: 2218-0532
Publisher: Österreichische Apotheker-Verlagsgesellschaft, m. b. H.

ARTICLE INFORMATION
==============================
DOI: 10.3797/scipharm.cespt.8.LDD06
Article ID: scipharm.2010.78.560
Subjects: Conference abstract LDD06

Delivering of Resveratrol with Solid Lipid Nanoparticles Improved Mitochondria Activity

Teskac K.
Pelipenko J.
Kristl J.
Faculty of Pharmacy, University of Ljubljana, Ljubljana, Slovenia
E-mail: julijana.kristl@ffa.uni-lj.si (J. Kristl)
Electronic publication date: 2010 Sep 30
Print publication date: 2010 Jul-Sep
Volume: 78
Issue: 3
First page: 560
Last page: 560
Copyright: © author(s); licensee Österreichische Apotheker-Verlagsgesellschaft m. b. H., Vienna, Austria.
Copyright year: 2010
License: This is an Open Access article distributed under the terms of the Creative Commons Attribution License (http://creativecommons.org/licenses/by/3.0/), which permits unrestricted use, distribution, and reproduction in any medium, provided the original work is properly cited.

JOURNAL INFORMATION
==============================
NLM Title Abbreviation: Sci Pharm
Journal ID: Sci Pharm
NLM Journal ID: 0026251
Journal ID: Scientia Pharmaceutica

Scientia Pharmaceutica

ISSN: 0036-8709
EISSN: 2218-0532
Publisher: Österreichische Apotheker-Verlagsgesellschaft, m. b. H.

ARTICLE INFORMATION
==============================
DOI: 10.3797/scipharm.cespt.8.LDD07
Article ID: scipharm.2010.78.561
Subjects: Conference abstract LDD07

Optimization of Cationic SLN for Gene Delivery

Doktorovova S. 1
Silva A. M. 2
Lopes C. M. 1 3
Martins-Lopes P. 1
Souto E. B. 1 3
1 Institute of Biotechnology and Bioengineering, Centre of Genomics and Biotechnology University of Trásos-Montes and Alto Douro (IBB/CGB-UTAD), Vila-Real, Portugal
2 Centre for the Research and Technology of Agro-Environmental and Biological Sciences, University of Trás-os-Montes and Alto Douro (CITAB-UTAD), Vila-Real, Portugal
3 Department of Pharmaceutical Technology, Faculty of Health Sciences, Fernando Pessoa University, Porto, Portugal
E-mails: d0450@utad.eu (S. Doktorovova), eliana@ufp.edu.pt (E. B.Souto)
Electronic publication date: 2010 Sep 30
Print publication date: 2010 Jul-Sep
Volume: 78
Issue: 3
First page: 561
Last page: 561
Copyright: © author(s); licensee Österreichische Apotheker-Verlagsgesellschaft m. b. H., Vienna, Austria.
Copyright year: 2010
License: This is an Open Access article distributed under the terms of the Creative Commons Attribution License (http://creativecommons.org/licenses/by/3.0/), which permits unrestricted use, distribution, and reproduction in any medium, provided the original work is properly cited.

The authors whish to thank Dr. Fernando Nunes for cooperation in freeze-drying experiments. This work was supported by grant SFRH/BD/60552/2009 from FCT and by Nadacia Slovenskeho Plynarenskeho Priemyslu.

JOURNAL INFORMATION
==============================
NLM Title Abbreviation: Sci Pharm
Journal ID: Sci Pharm
NLM Journal ID: 0026251
Journal ID: Scientia Pharmaceutica

Scientia Pharmaceutica

ISSN: 0036-8709
EISSN: 2218-0532
Publisher: Österreichische Apotheker-Verlagsgesellschaft, m. b. H.

ARTICLE INFORMATION
==============================
DOI: 10.3797/scipharm.cespt.8.LPES01
Article ID: scipharm.2010.78.562
Subjects: Conference abstract LPES01

The Potential of Continuous Processing in Secondary Manufacturing

Vangenechten R. 1
BACKX I. 2
1 Siemens Headquarters Pharma, Antwerp, Belgium
2 Siemens Industrial Automations and Druves Technology, Antwerp, Belgium
E-mail: rebecca.vangenechten@siemens.com (R. Vangenechten)
Electronic publication date: 2010 Sep 30
Print publication date: 2010 Jul-Sep
Volume: 78
Issue: 3
First page: 562
Last page: 562
Copyright: © author(s); licensee Österreichische Apotheker-Verlagsgesellschaft m. b. H., Vienna, Austria.
Copyright year: 2010
License: This is an Open Access article distributed under the terms of the Creative Commons Attribution License (http://creativecommons.org/licenses/by/3.0/), which permits unrestricted use, distribution, and reproduction in any medium, provided the original work is properly cited.

JOURNAL INFORMATION
==============================
NLM Title Abbreviation: Sci Pharm
Journal ID: Sci Pharm
NLM Journal ID: 0026251
Journal ID: Scientia Pharmaceutica

Scientia Pharmaceutica

ISSN: 0036-8709
EISSN: 2218-0532
Publisher: Österreichische Apotheker-Verlagsgesellschaft, m. b. H.

ARTICLE INFORMATION
==============================
DOI: 10.3797/scipharm.cespt.8.LPES02
Article ID: scipharm.2010.78.563
Subjects: Conference abstract LPES02

Measuring the Mixing Rate in Bladed Mixers

Radl S. 1 2
Brandl D. 1
Heimburg H. 1
Khinast J. G. 1 2
1 Graz University of Technology, Institute for Process and Particle Engineering, Graz, Austria
2 Research Center Pharmaceutical Engineering GmbH, Graz, Austria
E-mails: stefan.radl@tugraz.at (S. Radl), khinast@tugraz.at (J. G. Khinast)
Electronic publication date: 2010 Sep 30
Print publication date: 2010 Jul-Sep
Volume: 78
Issue: 3
First page: 563
Last page: 563
Copyright: © author(s); licensee Österreichische Apotheker-Verlagsgesellschaft m. b. H., Vienna, Austria.
Copyright year: 2010
License: This is an Open Access article distributed under the terms of the Creative Commons Attribution License (http://creativecommons.org/licenses/by/3.0/), which permits unrestricted use, distribution, and reproduction in any medium, provided the original work is properly cited.

JOURNAL INFORMATION
==============================
NLM Title Abbreviation: Sci Pharm
Journal ID: Sci Pharm
NLM Journal ID: 0026251
Journal ID: Scientia Pharmaceutica

Scientia Pharmaceutica

ISSN: 0036-8709
EISSN: 2218-0532
Publisher: Österreichische Apotheker-Verlagsgesellschaft, m. b. H.

ARTICLE INFORMATION
==============================
DOI: 10.3797/scipharm.cespt.8.LPES03
Article ID: scipharm.2010.78.564
Subjects: Conference abstract LPES03

LBM Simulation of Pharmaceutical Particles in Laminar Air Flows

Dietzel M.
Sommerfeld M.
Mechanische Verfahrenstechnik, MLU Halle-Wittenberg, Halle (Saale), Germany
E-mails: mathias.dietzel@iw.uni-halle.de (M. Dietzel), martin.sommerfeld@iw.uni-halle.de (M. Sommerfeld)
Electronic publication date: 2010 Sep 30
Print publication date: 2010 Jul-Sep
Volume: 78
Issue: 3
First page: 564
Last page: 564
Copyright: © author(s); licensee Österreichische Apotheker-Verlagsgesellschaft m. b. H., Vienna, Austria.
Copyright year: 2010
License: This is an Open Access article distributed under the terms of the Creative Commons Attribution License (http://creativecommons.org/licenses/by/3.0/), which permits unrestricted use, distribution, and reproduction in any medium, provided the original work is properly cited.

Fig. 1 Flow around a heterogeneous particle with shrinking second phase

JOURNAL INFORMATION
==============================
NLM Title Abbreviation: Sci Pharm
Journal ID: Sci Pharm
NLM Journal ID: 0026251
Journal ID: Scientia Pharmaceutica

Scientia Pharmaceutica

ISSN: 0036-8709
EISSN: 2218-0532
Publisher: Österreichische Apotheker-Verlagsgesellschaft, m. b. H.

ARTICLE INFORMATION
==============================
DOI: 10.3797/scipharm.cespt.8.LPES04
Article ID: scipharm.2010.78.565
Subjects: Conference abstract LPES04

Computational Fluid Dynamics, Sophisticated Marketing Tool or Reliable Method for Pharma- and Biotech Processes

Rinderhofer A. 1
Suzzi D. 2
Hörmann T. 2
Iannuccelli M. 2
Khinast J. G. 2 3
1 Zeta Biopharma GmbH, Tobelbad, Austria
2 Research Center Pharmaceutical Engineering GmbH, Graz, Austria
3 Graz University of Technology, Institute for Process and Particle Engineering, Graz, Austria
E-mails: alexander.rinderhofer@zeta.com (A. Rinderhofer), khinast@tugraz.at (J. G. Khinast)
Electronic publication date: 2010 Sep 30
Print publication date: 2010 Jul-Sep
Volume: 78
Issue: 3
First page: 565
Last page: 565
Copyright: © author(s); licensee Österreichische Apotheker-Verlagsgesellschaft m. b. H., Vienna, Austria.
Copyright year: 2010
License: This is an Open Access article distributed under the terms of the Creative Commons Attribution License (http://creativecommons.org/licenses/by/3.0/), which permits unrestricted use, distribution, and reproduction in any medium, provided the original work is properly cited.

JOURNAL INFORMATION
==============================
NLM Title Abbreviation: Sci Pharm
Journal ID: Sci Pharm
NLM Journal ID: 0026251
Journal ID: Scientia Pharmaceutica

Scientia Pharmaceutica

ISSN: 0036-8709
EISSN: 2218-0532
Publisher: Österreichische Apotheker-Verlagsgesellschaft, m. b. H.

ARTICLE INFORMATION
==============================
DOI: 10.3797/scipharm.cespt.8.LPES05
Article ID: scipharm.2010.78.566
Subjects: Conference abstract LPES05

Optimal Particle Size Distribution Control in a Pharmaceuticals Batch Dryer Using a Hybrid Genetic Algorithm-Pattern Search Optimization Strategy

Simon L. L. 1
Schongut M. 2
Stepanek F. 2
Hungerbuhler K. 1
1 ETH Zurich, Institute of Chemical and Bioengineering, Zurich, Switzerland
2 Institute of Chemical Technology, Department of Chemical Engineering, Prague, Czech Republic
E-mail: Levente.simon@chem.ethz.ch (L. L. Simon)
Electronic publication date: 2010 Sep 30
Print publication date: 2010 Jul-Sep
Volume: 78
Issue: 3
First page: 566
Last page: 566
Copyright: © author(s); licensee Österreichische Apotheker-Verlagsgesellschaft m. b. H., Vienna, Austria.
Copyright year: 2010
License: This is an Open Access article distributed under the terms of the Creative Commons Attribution License (http://creativecommons.org/licenses/by/3.0/), which permits unrestricted use, distribution, and reproduction in any medium, provided the original work is properly cited.

JOURNAL INFORMATION
==============================
NLM Title Abbreviation: Sci Pharm
Journal ID: Sci Pharm
NLM Journal ID: 0026251
Journal ID: Scientia Pharmaceutica

Scientia Pharmaceutica

ISSN: 0036-8709
EISSN: 2218-0532
Publisher: Österreichische Apotheker-Verlagsgesellschaft, m. b. H.

ARTICLE INFORMATION
==============================
DOI: 10.3797/scipharm.cespt.8.LPES06
Article ID: scipharm.2010.78.567
Subjects: Conference abstract LPES06

Combined Numerical Method for Multi-Scale Analysis of Tablet Coating Processes

Suzzi D. 1
Toschkoff G. 1
Machold D. 1
Radl S. 1 2
Khinast J. G. 1 2
1 Research Center Pharmaceutical Engineering, Graz, Austria
2 Institute for Process Engineering, Graz University of Technology, Graz, Austria
E-mails: daniele.suzzi@rcpe.at (D. Suzzi), khinast@tugraz.at (J. G. Khinast)
Electronic publication date: 2010 Sep 30
Print publication date: 2010 Jul-Sep
Volume: 78
Issue: 3
First page: 567
Last page: 567
Copyright: © author(s); licensee Österreichische Apotheker-Verlagsgesellschaft m. b. H., Vienna, Austria.
Copyright year: 2010
License: This is an Open Access article distributed under the terms of the Creative Commons Attribution License (http://creativecommons.org/licenses/by/3.0/), which permits unrestricted use, distribution, and reproduction in any medium, provided the original work is properly cited.

JOURNAL INFORMATION
==============================
NLM Title Abbreviation: Sci Pharm
Journal ID: Sci Pharm
NLM Journal ID: 0026251
Journal ID: Scientia Pharmaceutica

Scientia Pharmaceutica

ISSN: 0036-8709
EISSN: 2218-0532
Publisher: Österreichische Apotheker-Verlagsgesellschaft, m. b. H.

ARTICLE INFORMATION
==============================
DOI: 10.3797/scipharm.cespt.8.LPES07
Article ID: scipharm.2010.78.568
Subjects: Conference abstract LPES07

Numerical Simulation of Drop Impact on Wetted and Dry Surfaces

Gattringer M.
Steiner H.
Institute of Fluid mechanics and Heat transfer, Graz University of Technology, Graz, Austria
E-mail: helfried.steiner@tugraz.at (H. Steiner)
Electronic publication date: 2010 Sep 30
Print publication date: 2010 Jul-Sep
Volume: 78
Issue: 3
First page: 568
Last page: 568
Copyright: © author(s); licensee Österreichische Apotheker-Verlagsgesellschaft m. b. H., Vienna, Austria.
Copyright year: 2010
License: This is an Open Access article distributed under the terms of the Creative Commons Attribution License (http://creativecommons.org/licenses/by/3.0/), which permits unrestricted use, distribution, and reproduction in any medium, provided the original work is properly cited.

Fig. 1. Evolution of the liquid phase (denoted by red area) after drop impact on a wetted surface (We=203, Oh=0.005).

JOURNAL INFORMATION
==============================
NLM Title Abbreviation: Sci Pharm
Journal ID: Sci Pharm
NLM Journal ID: 0026251
Journal ID: Scientia Pharmaceutica

Scientia Pharmaceutica

ISSN: 0036-8709
EISSN: 2218-0532
Publisher: Österreichische Apotheker-Verlagsgesellschaft, m. b. H.

ARTICLE INFORMATION
==============================
DOI: 10.3797/scipharm.cespt.8.LNM01
Article ID: scipharm.2010.78.569
Subjects: Conference abstract LNM01

Magnetoliposomes: Liposomal Contrast Agents

Frascione D. 1
Saba-Lepek M. 1
Diwoky C. 2
Opriessnig P. 2
Stollberger R. 2
Almer G. 3
Mangge H. 3
Prassl R. 1
1 Institute of Biophysics and Nanosystems Research, Austrian Academy of Sciences, Graz, 8042, Austria
2 Institute of Medical Engineering, University of Technology, Graz, 8010, Austria
3 Medical University, Graz, 8010, Austria
E-mail: daniela.frascione@oeaw.ac.at (D. Frascione)
Electronic publication date: 2010 Sep 30
Print publication date: 2010 Jul-Sep
Volume: 78
Issue: 3
First page: 569
Last page: 569
Copyright: © author(s); licensee Österreichische Apotheker-Verlagsgesellschaft m. b. H., Vienna, Austria.
Copyright year: 2010
License: This is an Open Access article distributed under the terms of the Creative Commons Attribution License (http://creativecommons.org/licenses/by/3.0/), which permits unrestricted use, distribution, and reproduction in any medium, provided the original work is properly cited.

JOURNAL INFORMATION
==============================
NLM Title Abbreviation: Sci Pharm
Journal ID: Sci Pharm
NLM Journal ID: 0026251
Journal ID: Scientia Pharmaceutica

Scientia Pharmaceutica

ISSN: 0036-8709
EISSN: 2218-0532
Publisher: Österreichische Apotheker-Verlagsgesellschaft, m. b. H.

ARTICLE INFORMATION
==============================
DOI: 10.3797/scipharm.cespt.8.LNM02
Article ID: scipharm.2010.78.570
Subjects: Conference abstract LNM02

Determination of the structure of a new nanoscaled ultrasound contrast agent

Brüßler J. 1
Marxer E. E. J. 1
Becker A. 2
Schubert R. 3
Nimsky C. 2
Bakowsky U.
1 Department of Pharmaceutical Technology and Biopharmaceutics, Philipps-Universität, Marburg, Germany
2 Department of neuro surgery, Universitätsklinikum Gießen und Marburg GmbH, Marburg, Germany
3 Department of Pharmaceutical Technology and Biopharmaceutics, Albert-Ludwigs-Universität, Freiburg, Germany
E-mail: jana.bruessler@staff.uni-marburg.de (J. Brüßler)
Electronic publication date: 2010 Sep 30
Print publication date: 2010 Jul-Sep
Volume: 78
Issue: 3
First page: 570
Last page: 570
Copyright: © author(s); licensee Österreichische Apotheker-Verlagsgesellschaft m. b. H., Vienna, Austria.
Copyright year: 2010
License: This is an Open Access article distributed under the terms of the Creative Commons Attribution License (http://creativecommons.org/licenses/by/3.0/), which permits unrestricted use, distribution, and reproduction in any medium, provided the original work is properly cited.

The authors would like to thank the DFG “Forschergruppe Nanohale 695” for support.

JOURNAL INFORMATION
==============================
NLM Title Abbreviation: Sci Pharm
Journal ID: Sci Pharm
NLM Journal ID: 0026251
Journal ID: Scientia Pharmaceutica

Scientia Pharmaceutica

ISSN: 0036-8709
EISSN: 2218-0532
Publisher: Österreichische Apotheker-Verlagsgesellschaft, m. b. H.

ARTICLE INFORMATION
==============================
DOI: 10.3797/scipharm.cespt.8.LNM03
Article ID: scipharm.2010.78.571
Subjects: Conference abstract LNM03

Effect of High-Pressure Homogenization on the Formulation of Micro- and Nanocrystals Containing Poorly Watersoluble Meloxicam

Ambrus R. 1
Pomázi A. 1
Kristl J. 2
Kocbek P. 2
Szabó-révész P. 1
1 Department of Pharmaceutical Technology, University of Szeged, Szeged, Hungary
2 Faculty of Pharmacy, University of Ljubljana, Ljubljana, Slovenia
E-mail: arita@pharm.u-szeged.hu (R. Ambrus)
Electronic publication date: 2010 Sep 30
Print publication date: 2010 Jul-Sep
Volume: 78
Issue: 3
First page: 571
Last page: 571
Copyright: © author(s); licensee Österreichische Apotheker-Verlagsgesellschaft m. b. H., Vienna, Austria.
Copyright year: 2010
License: This is an Open Access article distributed under the terms of the Creative Commons Attribution License (http://creativecommons.org/licenses/by/3.0/), which permits unrestricted use, distribution, and reproduction in any medium, provided the original work is properly cited.

JOURNAL INFORMATION
==============================
NLM Title Abbreviation: Sci Pharm
Journal ID: Sci Pharm
NLM Journal ID: 0026251
Journal ID: Scientia Pharmaceutica

Scientia Pharmaceutica

ISSN: 0036-8709
EISSN: 2218-0532
Publisher: Österreichische Apotheker-Verlagsgesellschaft, m. b. H.

ARTICLE INFORMATION
==============================
DOI: 10.3797/scipharm.cespt.8.LPAT01
Article ID: scipharm.2010.78.572
Subjects: Conference abstract LPAT01

Real Time Measurement of Particle Size as a Response to the FDA’s PAT Regulatory Framework

Looser B. 1
Vaisman A. 2
Blasco A. 3
1 Malvern Instruments, Malvern, United Kingdom
2 Malvern Instruments, Westborough, MA, USA
3 Malvern Instruments, Orsay, France
E-mail: Bernd.Looser@malvern.com (B. Looser), Alon.Vaisman@malvern.com (A. Vaisman), Alain.Blasco@malvern.com (A. Blasco)
Electronic publication date: 2010 Sep 30
Print publication date: 2010 Jul-Sep
Volume: 78
Issue: 3
First page: 572
Last page: 572
Copyright: © author(s); licensee Österreichische Apotheker-Verlagsgesellschaft m. b. H., Vienna, Austria.
Copyright year: 2010
License: This is an Open Access article distributed under the terms of the Creative Commons Attribution License (http://creativecommons.org/licenses/by/3.0/), which permits unrestricted use, distribution, and reproduction in any medium, provided the original work is properly cited.

JOURNAL INFORMATION
==============================
NLM Title Abbreviation: Sci Pharm
Journal ID: Sci Pharm
NLM Journal ID: 0026251
Journal ID: Scientia Pharmaceutica

Scientia Pharmaceutica

ISSN: 0036-8709
EISSN: 2218-0532
Publisher: Österreichische Apotheker-Verlagsgesellschaft, m. b. H.

ARTICLE INFORMATION
==============================
DOI: 10.3797/scipharm.cespt.8.LPAT02
Article ID: scipharm.2010.78.573
Subjects: Conference abstract LPAT02

Real-Time Monitoring of Powder Mixing Dynamics with Spectroscopic PAT-Tools

Koller D. M. 1
Balak N. 1
Scheibelhofer O. 1
Moor J. 1
Hörl G. 1
Heigl N. 1
Fraser S. D. 1
Khinast J. G. 1 2
1 Research Center Pharmaceutical Engineering GmbH, Graz, Austria
2 Institute for Process and Particle Engineering, Graz University of Technology, Graz, Austria
E-mail: daniel.koller@rcpe.at (D. M. Koller), khinast@tugraz.at (J. G. Khinast)
Electronic publication date: 2010 Sep 30
Print publication date: 2010 Jul-Sep
Volume: 78
Issue: 3
First page: 573
Last page: 573
Copyright: © author(s); licensee Österreichische Apotheker-Verlagsgesellschaft m. b. H., Vienna, Austria.
Copyright year: 2010
License: This is an Open Access article distributed under the terms of the Creative Commons Attribution License (http://creativecommons.org/licenses/by/3.0/), which permits unrestricted use, distribution, and reproduction in any medium, provided the original work is properly cited.

JOURNAL INFORMATION
==============================
NLM Title Abbreviation: Sci Pharm
Journal ID: Sci Pharm
NLM Journal ID: 0026251
Journal ID: Scientia Pharmaceutica

Scientia Pharmaceutica

ISSN: 0036-8709
EISSN: 2218-0532
Publisher: Österreichische Apotheker-Verlagsgesellschaft, m. b. H.

ARTICLE INFORMATION
==============================
DOI: 10.3797/scipharm.cespt.8.LPAT03
Article ID: scipharm.2010.78.574
Subjects: Conference abstract LPAT03

A Contribution to the Application of Near-Infrared Spectroscopy for Tablet Analysis: Metformin Hydrochloride Tablets Case Study

Marić L.
Homšek I.
R&D Institute, Galenika a.d., Batajnički drum b.b., 11080 Belgrade, Serbia
E-mails: razumpolze@gmail.com (L.Marić), irena.homsek@gmail.com (I. Homšek)
Electronic publication date: 2010 Sep 30
Print publication date: 2010 Jul-Sep
Volume: 78
Issue: 3
First page: 574
Last page: 574
Copyright: © author(s); licensee Österreichische Apotheker-Verlagsgesellschaft m. b. H., Vienna, Austria.
Copyright year: 2010
License: This is an Open Access article distributed under the terms of the Creative Commons Attribution License (http://creativecommons.org/licenses/by/3.0/), which permits unrestricted use, distribution, and reproduction in any medium, provided the original work is properly cited.

JOURNAL INFORMATION
==============================
NLM Title Abbreviation: Sci Pharm
Journal ID: Sci Pharm
NLM Journal ID: 0026251
Journal ID: Scientia Pharmaceutica

Scientia Pharmaceutica

ISSN: 0036-8709
EISSN: 2218-0532
Publisher: Österreichische Apotheker-Verlagsgesellschaft, m. b. H.

ARTICLE INFORMATION
==============================
DOI: 10.3797/scipharm.cespt.8.LNT01
Article ID: scipharm.2010.78.575
Subjects: Conference abstract LNT01

Important Parameters in Cytotoxicity Testing of Nanoparticles

Meindl C. 1 2
Roblegg E. 3
Pieber T. R. 2
Fröhlich E. 1 2
1 Center for Medical Research, Medical University of Graz, Austria
2 Department of Internal Medicine, Division of Endocrinology and Nuclear Medicine, Medical University of Graz, Austria
3 Institute of Pharmaceutical Sciences, Department of Pharmaceutical Technology, Karl-Franzens-University of Graz, Austria
E-mail: eleonore.froehlich@medunigraz.at (E. Fröhlich)
Electronic publication date: 2010 Sep 30
Print publication date: 2010 Jul-Sep
Volume: 78
Issue: 3
First page: 575
Last page: 575
Copyright: © author(s); licensee Österreichische Apotheker-Verlagsgesellschaft m. b. H., Vienna, Austria.
Copyright year: 2010
License: This is an Open Access article distributed under the terms of the Creative Commons Attribution License (http://creativecommons.org/licenses/by/3.0/), which permits unrestricted use, distribution, and reproduction in any medium, provided the original work is properly cited.

JOURNAL INFORMATION
==============================
NLM Title Abbreviation: Sci Pharm
Journal ID: Sci Pharm
NLM Journal ID: 0026251
Journal ID: Scientia Pharmaceutica

Scientia Pharmaceutica

ISSN: 0036-8709
EISSN: 2218-0532
Publisher: Österreichische Apotheker-Verlagsgesellschaft, m. b. H.

ARTICLE INFORMATION
==============================
DOI: 10.3797/scipharm.cespt.8.LNT02
Article ID: scipharm.2010.78.576
Subjects: Conference abstract LNT02

Development of Solid Lipid Nanoparticles for Praziquantel Delivery: Particle Size Characterization and Cell Toxicity Assessment

Souza A. L. R. 1 2
Andreani T. 2
Doktorovová S. 3
Silva A. M. 2 4
Souto E. B. 5
Gremião M. P. D. 1
1 Department of Pharmaceutical Technology, Faculty of Pharmaceutical Sciences, UNESP, Araraquara, Brazil
2 Department of Biology and Environment, University of Trás-os-Montes and Alto Douro, Vila Real, Portugal
3 Centre of Genomics and Biotechnology, IBB/CGB-UTAD, Vila-Real, Portugal
4 Centre for Research and Technology of Agro-Environmental and Biological Sciences, Vila Real, Portugal
5 Department of Pharmaceutical Technology, Faculty of Health Sciences, UFP, Porto, Portugal
E-mail: analuizars@yahoo.com.br (A. L. R. Souza)
Electronic publication date: 2010 Sep 30
Print publication date: 2010 Jul-Sep
Volume: 78
Issue: 3
First page: 576
Last page: 576
Copyright: © author(s); licensee Österreichische Apotheker-Verlagsgesellschaft m. b. H., Vienna, Austria.
Copyright year: 2010
License: This is an Open Access article distributed under the terms of the Creative Commons Attribution License (http://creativecommons.org/licenses/by/3.0/), which permits unrestricted use, distribution, and reproduction in any medium, provided the original work is properly cited.

JOURNAL INFORMATION
==============================
NLM Title Abbreviation: Sci Pharm
Journal ID: Sci Pharm
NLM Journal ID: 0026251
Journal ID: Scientia Pharmaceutica

Scientia Pharmaceutica

ISSN: 0036-8709
EISSN: 2218-0532
Publisher: Österreichische Apotheker-Verlagsgesellschaft, m. b. H.

ARTICLE INFORMATION
==============================
DOI: 10.3797/scipharm.cespt.8.LNT03
Article ID: scipharm.2010.78.577
Subjects: Conference abstract LNT03

Does Continuous Exposure to ZnO and TiO2 NPs Cause any Changes in Keratinocytes?

Kocbek P. 1
Teskač K. 1
Kreft M. E. 2
Kristl J. 1
1 Faculty of Pharmacy, University of Ljubljana, Ljubljana, Slovenia
2 Institute of Cell Biology, Faculty of Medicine, University of Ljubljana, Ljubljana, Slovenia
E-mail: petra.kocbek@ffa.uni-lj.si (P. Kocbek)
Electronic publication date: 2010 Sep 30
Print publication date: 2010 Jul-Sep
Volume: 78
Issue: 3
First page: 577
Last page: 577
Copyright: © author(s); licensee Österreichische Apotheker-Verlagsgesellschaft m. b. H., Vienna, Austria.
Copyright year: 2010
License: This is an Open Access article distributed under the terms of the Creative Commons Attribution License (http://creativecommons.org/licenses/by/3.0/), which permits unrestricted use, distribution, and reproduction in any medium, provided the original work is properly cited.

JOURNAL INFORMATION
==============================
NLM Title Abbreviation: Sci Pharm
Journal ID: Sci Pharm
NLM Journal ID: 0026251
Journal ID: Scientia Pharmaceutica

Scientia Pharmaceutica

ISSN: 0036-8709
EISSN: 2218-0532
Publisher: Österreichische Apotheker-Verlagsgesellschaft, m. b. H.

ARTICLE INFORMATION
==============================
DOI: 10.3797/scipharm.cespt.8.LNT04
Article ID: scipharm.2010.78.578
Subjects: Conference abstract LNT04

Transport Studies of Nanostructured Materials across the Buccal Mucosa

Roblegg E. 1
Fröhlich E. 2
Leitinger G. 2
Letofsky-Papst I. 3
Zimmer A. 1
1 Institute of Pharmaceutical Sciences/ Pharmaceutical Technology, Karl-Franzens University Graz, Austria
2 Center for Medical Research, Medical University of Graz, Austria
3 Research Institute for Electron Microscopy, University of Technology Graz, Austria
E-mail: eva.roblegg@uni-graz.at (E. Roblegg)
Electronic publication date: 2010 Sep 30
Print publication date: 2010 Jul-Sep
Volume: 78
Issue: 3
First page: 578
Last page: 578
Copyright: © author(s); licensee Österreichische Apotheker-Verlagsgesellschaft m. b. H., Vienna, Austria.
Copyright year: 2010
License: This is an Open Access article distributed under the terms of the Creative Commons Attribution License (http://creativecommons.org/licenses/by/3.0/), which permits unrestricted use, distribution, and reproduction in any medium, provided the original work is properly cited.

JOURNAL INFORMATION
==============================
NLM Title Abbreviation: Sci Pharm
Journal ID: Sci Pharm
NLM Journal ID: 0026251
Journal ID: Scientia Pharmaceutica

Scientia Pharmaceutica

ISSN: 0036-8709
EISSN: 2218-0532
Publisher: Österreichische Apotheker-Verlagsgesellschaft, m. b. H.

ARTICLE INFORMATION
==============================
DOI: 10.3797/scipharm.cespt.8.LNT05
Article ID: scipharm.2010.78.579
Subjects: Conference abstract LNT05

A Model for Studying Nasal Drug Delivery: RPMI 2650 Human Nasal Epithelial Cell Line

Kürti L. 1 2
Kis L. 1 2
Veszelka S. 2
Szabó-Révész P. 1
Deli M. A. 2
1 Department of Pharmaceutical Technology, University of Szeged, Szeged, Hungary
2 Laboratory of Molecular Neurobiology, Institute of Biophysics, Biological Research Center, Hungarian Academy of Sciences, Szeged, Hungary
E-mail: leventekurti@pharm.u-szeged.hu (L.Kürti)
Electronic publication date: 2010 Sep 30
Print publication date: 2010 Jul-Sep
Volume: 78
Issue: 3
First page: 579
Last page: 579
Copyright: © author(s); licensee Österreichische Apotheker-Verlagsgesellschaft m. b. H., Vienna, Austria.
Copyright year: 2010
License: This is an Open Access article distributed under the terms of the Creative Commons Attribution License (http://creativecommons.org/licenses/by/3.0/), which permits unrestricted use, distribution, and reproduction in any medium, provided the original work is properly cited.

JOURNAL INFORMATION
==============================
NLM Title Abbreviation: Sci Pharm
Journal ID: Sci Pharm
NLM Journal ID: 0026251
Journal ID: Scientia Pharmaceutica

Scientia Pharmaceutica

ISSN: 0036-8709
EISSN: 2218-0532
Publisher: Österreichische Apotheker-Verlagsgesellschaft, m. b. H.

ARTICLE INFORMATION
==============================
DOI: 10.3797/scipharm.cespt.8.LNT06
Article ID: scipharm.2010.78.580
Subjects: Conference abstract LNT06

European Center for Nanotoxicology – Nanoscale Materials: A New Challenge for Toxicology

Sinner F. 1 2
Roblegg E. 3
Fröhlich E. 4
Zimmer A. 3
Falk A. 1
1 BioNanoNet Forschungsgesellschaft mbH, Graz, Austria
2 Health – Institute of Biomedicine and Health Sciences, Joanneum Research Graz, Austria
3 Institute of Pharmaceutical Sciences/ Pharmaceutical Technology, Karl-Franzens University Graz, Austria
4 Center for Medical Research, Medical University of Graz, Austria
E-mail: frank.sinner@bionanonet.at (F. Sinner)
Electronic publication date: 2010 Sep 30
Print publication date: 2010 Jul-Sep
Volume: 78
Issue: 3
First page: 580
Last page: 580
Copyright: © author(s); licensee Österreichische Apotheker-Verlagsgesellschaft m. b. H., Vienna, Austria.
Copyright year: 2010
License: This is an Open Access article distributed under the terms of the Creative Commons Attribution License (http://creativecommons.org/licenses/by/3.0/), which permits unrestricted use, distribution, and reproduction in any medium, provided the original work is properly cited.

JOURNAL INFORMATION
==============================
NLM Title Abbreviation: Sci Pharm
Journal ID: Sci Pharm
NLM Journal ID: 0026251
Journal ID: Scientia Pharmaceutica

Scientia Pharmaceutica

ISSN: 0036-8709
EISSN: 2218-0532
Publisher: Österreichische Apotheker-Verlagsgesellschaft, m. b. H.

ARTICLE INFORMATION
==============================
DOI: 10.3797/scipharm.cespt.8.LPPT01
Article ID: scipharm.2010.78.581
Subjects: Conference abstract LPPT01

Quantitative Design of Continuous Mixing Processes for Pharmaceutical Applications

Muzzio F.
Rutgers, The State University of New Jersey, USA
E-mail: fjmuzzio@yahoo.com
Electronic publication date: 2010 Sep 30
Print publication date: 2010 Jul-Sep
Volume: 78
Issue: 3
First page: 581
Last page: 581
Copyright: © author(s); licensee Österreichische Apotheker-Verlagsgesellschaft m. b. H., Vienna, Austria.
Copyright year: 2010
License: This is an Open Access article distributed under the terms of the Creative Commons Attribution License (http://creativecommons.org/licenses/by/3.0/), which permits unrestricted use, distribution, and reproduction in any medium, provided the original work is properly cited.

JOURNAL INFORMATION
==============================
NLM Title Abbreviation: Sci Pharm
Journal ID: Sci Pharm
NLM Journal ID: 0026251
Journal ID: Scientia Pharmaceutica

Scientia Pharmaceutica

ISSN: 0036-8709
EISSN: 2218-0532
Publisher: Österreichische Apotheker-Verlagsgesellschaft, m. b. H.

ARTICLE INFORMATION
==============================
DOI: 10.3797/scipharm.cespt.8.LPPT02
Article ID: scipharm.2010.78.582
Subjects: Conference abstract LPPT02

Do the Characteristics of Xanthan Single Molecule Reflect the Behavior of Matrix Tablets?

Baumgartner S. 1
Kristl J. 1
Govedarica B. 1
Srčič S. 1
Valant A. Zupančič 2
Mikac U. 3
Sepe A. 3
1 University of Ljubljana, Faculty of Pharmacy, Ljubljana, Slovenia
2 University of Ljubljana, Faculty of Chemistry and Chemical Technology, Ljubljana, Slovenia
3 Jožef Stefan Institute, Ljubljana, Slovenia
E-mail: sasa.baumgartner@ffa.uni-lj.si (S. Baumgartner)
Electronic publication date: 2010 Sep 30
Print publication date: 2010 Jul-Sep
Volume: 78
Issue: 3
First page: 582
Last page: 582
Copyright: © author(s); licensee Österreichische Apotheker-Verlagsgesellschaft m. b. H., Vienna, Austria.
Copyright year: 2010
License: This is an Open Access article distributed under the terms of the Creative Commons Attribution License (http://creativecommons.org/licenses/by/3.0/), which permits unrestricted use, distribution, and reproduction in any medium, provided the original work is properly cited.

JOURNAL INFORMATION
==============================
NLM Title Abbreviation: Sci Pharm
Journal ID: Sci Pharm
NLM Journal ID: 0026251
Journal ID: Scientia Pharmaceutica

Scientia Pharmaceutica

ISSN: 0036-8709
EISSN: 2218-0532
Publisher: Österreichische Apotheker-Verlagsgesellschaft, m. b. H.

ARTICLE INFORMATION
==============================
DOI: 10.3797/scipharm.cespt.8.LPPT03
Article ID: scipharm.2010.78.583
Subjects: Conference abstract LPPT03

Development of Modified Release Pellets via Dry Powder Coating

Smikalla M. 1
Urbanetz N. A. 2
1 Institute of Pharmaceutics and Biopharmaceutics, Heinrich-Heine-University, Duesseldorf, Germany
2 Research Center Pharmaceutical Engineering, Graz University of Technology, Graz, Austria
E-mail: martina.smikalla@uni-duesseldorf.de (M. Smikalla)
Electronic publication date: 2010 Sep 30
Print publication date: 2010 Jul-Sep
Volume: 78
Issue: 3
First page: 583
Last page: 583
Copyright: © author(s); licensee Österreichische Apotheker-Verlagsgesellschaft m. b. H., Vienna, Austria.
Copyright year: 2010
License: This is an Open Access article distributed under the terms of the Creative Commons Attribution License (http://creativecommons.org/licenses/by/3.0/), which permits unrestricted use, distribution, and reproduction in any medium, provided the original work is properly cited.

JOURNAL INFORMATION
==============================
NLM Title Abbreviation: Sci Pharm
Journal ID: Sci Pharm
NLM Journal ID: 0026251
Journal ID: Scientia Pharmaceutica

Scientia Pharmaceutica

ISSN: 0036-8709
EISSN: 2218-0532
Publisher: Österreichische Apotheker-Verlagsgesellschaft, m. b. H.

ARTICLE INFORMATION
==============================
DOI: 10.3797/scipharm.cespt.8.LPPT04
Article ID: scipharm.2010.78.584
Subjects: Conference abstract LPPT04

Effect of Inert Core Material on the Processability and Quality Characteristics of Coated Pellets

Antal I. 1
Kállai N. 1
Luhn O. 2
Mola J. H. 1
Ruszkai A. 1
Balogh E. 1
Dredán 1
Klebovich I. 1
1 Department of Pharmaceutics, Semmelweis University, Budapest, Hungary
2 BENEO-Palatinit GmbH, Mannheim, Germany
E-mail: antist@gyok.sote.hu (I. Antal)
Electronic publication date: 2010 Sep 30
Print publication date: 2010 Jul-Sep
Volume: 78
Issue: 3
First page: 584
Last page: 584
Copyright: © author(s); licensee Österreichische Apotheker-Verlagsgesellschaft m. b. H., Vienna, Austria.
Copyright year: 2010
License: This is an Open Access article distributed under the terms of the Creative Commons Attribution License (http://creativecommons.org/licenses/by/3.0/), which permits unrestricted use, distribution, and reproduction in any medium, provided the original work is properly cited.

JOURNAL INFORMATION
==============================
NLM Title Abbreviation: Sci Pharm
Journal ID: Sci Pharm
NLM Journal ID: 0026251
Journal ID: Scientia Pharmaceutica

Scientia Pharmaceutica

ISSN: 0036-8709
EISSN: 2218-0532
Publisher: Österreichische Apotheker-Verlagsgesellschaft, m. b. H.

ARTICLE INFORMATION
==============================
DOI: 10.3797/scipharm.cespt.8.LPPT05
Article ID: scipharm.2010.78.585
Subjects: Conference abstract LPPT05

Pharmaceutical Hot Melt Extrusion

Neub N.
Coperion GmbH, Theodorstraße 10, 70469 Stuttgart, Germany
E-mail: nicole.neub@coperion.com
Electronic publication date: 2010 Sep 30
Print publication date: 2010 Jul-Sep
Volume: 78
Issue: 3
First page: 585
Last page: 585
Copyright: © author(s); licensee Österreichische Apotheker-Verlagsgesellschaft m. b. H., Vienna, Austria.
Copyright year: 2010
License: This is an Open Access article distributed under the terms of the Creative Commons Attribution License (http://creativecommons.org/licenses/by/3.0/), which permits unrestricted use, distribution, and reproduction in any medium, provided the original work is properly cited.

JOURNAL INFORMATION
==============================
NLM Title Abbreviation: Sci Pharm
Journal ID: Sci Pharm
NLM Journal ID: 0026251
Journal ID: Scientia Pharmaceutica

Scientia Pharmaceutica

ISSN: 0036-8709
EISSN: 2218-0532
Publisher: Österreichische Apotheker-Verlagsgesellschaft, m. b. H.

ARTICLE INFORMATION
==============================
DOI: 10.3797/scipharm.cespt.8.LPPT06
Article ID: scipharm.2010.78.586
Subjects: Conference abstract LPPT06

Spatial Filtering Technique as Powerful Tool for Real-Time Particle Size Measurement for Fluid Bed Applications in Pharmaceutical Industry

Dietrich S. 1
Petrak D. 2
Köhler M. 1
Eckardt G. 1
1 Parsum GmbH, Chemnitz, Germany
2 Chemnitz University of Technology, Chemnitz, Germany
E-mail: petrak@imech.tu-chemnitz.de (D. Petrak)
Electronic publication date: 2010 Sep 30
Print publication date: 2010 Jul-Sep
Volume: 78
Issue: 3
First page: 586
Last page: 586
Copyright: © author(s); licensee Österreichische Apotheker-Verlagsgesellschaft m. b. H., Vienna, Austria.
Copyright year: 2010
License: This is an Open Access article distributed under the terms of the Creative Commons Attribution License (http://creativecommons.org/licenses/by/3.0/), which permits unrestricted use, distribution, and reproduction in any medium, provided the original work is properly cited.

JOURNAL INFORMATION
==============================
NLM Title Abbreviation: Sci Pharm
Journal ID: Sci Pharm
NLM Journal ID: 0026251
Journal ID: Scientia Pharmaceutica

Scientia Pharmaceutica

ISSN: 0036-8709
EISSN: 2218-0532
Publisher: Österreichische Apotheker-Verlagsgesellschaft, m. b. H.

ARTICLE INFORMATION
==============================
DOI: 10.3797/scipharm.cespt.8.LPPT07
Article ID: scipharm.2010.78.587
Subjects: Conference abstract LPPT07

Evaluation of Micro Fluidic High Pressure Dispersion/Emulsification for SLN Production

Finke J. H. 1
Schur J. 1
Gothsch T. 2
Beinert S. 2
Lesche C. 3
Büttgenbach S. 3
Kwade A. 2
Müller-Goymann C. C. 1
1 Institut für Pharmazeutische Technologie, Technische Universität Braunschweig, Braunschweig, Germany
2 Institut für Partikeltechnik, Technische Universität Braunschweig, Braunschweig, Germany
3 Institut für Mikrotechnik, Technische Universität Braunschweig, Braunschweig, Germany
E-mails: jan.finke@tu-bs.de (J. H. Finke), c.mueller-goymann@tu-bs.de (C. C. Müller-Goymann)
Electronic publication date: 2010 Sep 30
Print publication date: 2010 Jul-Sep
Volume: 78
Issue: 3
First page: 587
Last page: 587
Copyright: © author(s); licensee Österreichische Apotheker-Verlagsgesellschaft m. b. H., Vienna, Austria.
Copyright year: 2010
License: This is an Open Access article distributed under the terms of the Creative Commons Attribution License (http://creativecommons.org/licenses/by/3.0/), which permits unrestricted use, distribution, and reproduction in any medium, provided the original work is properly cited.

The authors gratefully acknowledge the DFG for financial support of the DFG research group 856 “Mikrosysteme für partikuläre Life-Science-Produkte“ ().

JOURNAL INFORMATION
==============================
NLM Title Abbreviation: Sci Pharm
Journal ID: Sci Pharm
NLM Journal ID: 0026251
Journal ID: Scientia Pharmaceutica

Scientia Pharmaceutica

ISSN: 0036-8709
EISSN: 2218-0532
Publisher: Österreichische Apotheker-Verlagsgesellschaft, m. b. H.

ARTICLE INFORMATION
==============================
DOI: 10.3797/scipharm.cespt.8.LPM01
Article ID: scipharm.2010.78.588
Subjects: Conference abstract LPM01

Iterative Method for Constructing Dosing Regimes for Controlling HIV Dynamics

Evans N. D.
School of Engineering, University of Warwick, Coventry, CV4 7AL, United Kingdom
E-mail: Neil.Evans@warwick.ac.uk
Electronic publication date: 2010 Sep 30
Print publication date: 2010 Jul-Sep
Volume: 78
Issue: 3
First page: 588
Last page: 588
Copyright: © author(s); licensee Österreichische Apotheker-Verlagsgesellschaft m. b. H., Vienna, Austria.
Copyright year: 2010
License: This is an Open Access article distributed under the terms of the Creative Commons Attribution License (http://creativecommons.org/licenses/by/3.0/), which permits unrestricted use, distribution, and reproduction in any medium, provided the original work is properly cited.

JOURNAL INFORMATION
==============================
NLM Title Abbreviation: Sci Pharm
Journal ID: Sci Pharm
NLM Journal ID: 0026251
Journal ID: Scientia Pharmaceutica

Scientia Pharmaceutica

ISSN: 0036-8709
EISSN: 2218-0532
Publisher: Österreichische Apotheker-Verlagsgesellschaft, m. b. H.

ARTICLE INFORMATION
==============================
DOI: 10.3797/scipharm.cespt.8.LPM02
Article ID: scipharm.2010.78.589
Subjects: Conference abstract LPM02

Alternatives to Laboratory Animals: In Vitro and In Silico Approaches

Cascone S. 1
De Santis F. 1
Lamberti G. 1
Titomanlio G. 1
Barba A. A. 2
1 Dipartimento di Ingegneria Chimica e Alimentare, Università di Salerno, Fisciano, Italia
2 Dipartimento di Scienze Farmaceutiche, Università di Salerno, Fisciano, Italia
E-mails: scascone@unisa.it (S. Cascone), fedesantis@unisa.it (F. De Santis), glamberti@unisa.it (G. Lamberti), gtitomanlio@unisa.it (G. Titomanlio), aabarba@unisa.it (A. A. Barba)
Electronic publication date: 2010 Sep 30
Print publication date: 2010 Jul-Sep
Volume: 78
Issue: 3
First page: 589
Last page: 589
Copyright: © author(s); licensee Österreichische Apotheker-Verlagsgesellschaft m. b. H., Vienna, Austria.
Copyright year: 2010
License: This is an Open Access article distributed under the terms of the Creative Commons Attribution License (http://creativecommons.org/licenses/by/3.0/), which permits unrestricted use, distribution, and reproduction in any medium, provided the original work is properly cited.

Fig. 1 Haematic drug concentration after the administration from three solid dosage forms (fast, medium and slow release). Symbols: experimental data, curves: model prediction.

This work was supported by a scholarship from the Austrian Federal Ministry for Education, Science, and Culture to RM.

This work was supported by Deutsche Forschungsgemeinschaft (DFG grant SO 204/36-1, SPP 1423).

This work was supported by TÁMOP-Hungary research project: Development of teranostics in cardiovascular, metabolics, and inflammatory diseases (TÁMOP-4.2.2-08/1-2008-0013).

This work was supported by the FP6 European integrated project “NanoBioPharmaceutics”, NMP4-CT-2006-026723 and by the RPC project ‘Nano-structured Materials for Drug Targeting, Release and Imaging’.

This work was supported by CAPES.

This work was supported by TÁMOP-Hungary research project: Development of teranostics in cardiovascular, metabolic, and inflammatory diseases (TÁMOP-4.2.2-08/1-2008-0013).

